# Protective Efficacy of Intermittent Preventive Treatment of Malaria in Infants (IPTi) Using Sulfadoxine-Pyrimethamine and Parasite Resistance

**DOI:** 10.1371/journal.pone.0012618

**Published:** 2010-09-07

**Authors:** Jamie T. Griffin, Matthew Cairns, Azra C. Ghani, Cally Roper, David Schellenberg, Ilona Carneiro, Robert D. Newman, Martin P. Grobusch, Brian Greenwood, Daniel Chandramohan, Roly D. Gosling

**Affiliations:** 1 MRC Centre for Outbreak Analysis and Modelling, Department of Infectious Disease Epidemiology, Imperial College, London, United Kingdom; 2 Department of Infectious and Tropical Diseases, London School of Hygiene & Tropical Medicine, London, United Kingdom; 3 Malaria Branch, Centers for Disease Control and Prevention, Atlanta, Georgia, United States of America; 4 Department of Internal Medicine, Division of Infectious Diseases, Tropical Medicine and AIDS, Amsterdam Medical Center, University of Amsterdam, Amsterdam, The Netherlands; 5 Medical Research Unit, Hospital Albert Schweitzer, Lambaréné, Gabon; Wellcome Trust Mahidol University-Oxford Tropical Medicine Research Unit (MORU), Thailand

## Abstract

**Background:**

Intermittent Preventive Treatment of malaria in infants using sulfadoxine-pyrimethamine (SP-IPTi) is recommended by WHO for implementation in settings where resistance to SP is not high. Here we examine the relationship between the protective efficacy of SP-IPTi and measures of SP resistance.

**Methods and Results:**

We analysed the relationship between protective efficacy reported in the 7 SP-IPTi trials and contemporaneous data from 6 *in vivo* efficacy studies using SP and 7 molecular studies reporting frequency of *dhfr* triple and *dhps* double mutations within 50km of the trial sites. We found a borderline significant association between frequency of the *dhfr* triple mutation and protective efficacy to 12 months of age of SP-IPTi. This association is significantly biased due to differences between studies, namely number of doses of SP given and follow up times. However, fitting a simple probabilistic model to determine the relationship between the frequency of the *dhfr* triple, *dhps* double and *dhfr/dhps* quintuple mutations associated with resistance to SP and protective efficacy, we found a significant inverse relationship between the *dhfr* triple mutation frequency alone and the *dhfr/dhps* quintuple mutations and efficacy at 35 days post the 9 month dose and up to 12 months of age respectively.

**Conclusions:**

A significant relationship was found between the frequency of the *dhfr* triple mutation and SP-IPTi protective efficacy at 35 days post the 9 month dose. An association between the protective efficacy to 12 months of age and *dhfr* triple and *dhfr/dhps* quintuple mutations was found but should be viewed with caution due to bias. It was not possible to define a more definite relationship based on the data available from these trials.

## Introduction

Intermittent preventive treatment of malaria with sulfadoxine-pyrimethamine (SP) in infants (SP- IPTi) reduced the incidence of clinical malaria in areas of sub-Saharan Africa with low to moderate SP resistance in sub-Saharan Africa [1]–[5] but had no significant protective effect in one area of high SP resistance [6] and one area of low transmission [7]. Based on the findings from these 7 randomised trials, a technical Expert Group convened by the World Health Organization (WHO) in 2009 recommended SP-IPTi for use as a malaria control tool in sub-Saharan Africa under certain conditions [8]. Firstly, it was recommended that IPTi programmes be implemented only in areas with moderate to high transmission (Annual Entomological Inoculation Rates (EIR) greater than 10 infectious bites per person per year). Second, it was recommended that programmes are not implemented in areas where the degree of parasite resistance to SP is high. At that time the relationship between the level of SP resistance and the likely efficacy of SP-IPTi at any individual site was not well defined. The aim of this study was to explore the relationship between protective efficacy of SP-IPTi and measures of resistance to SP in order to better define this relationship.

The most common method for estimating SP resistance is measurement of the *in vivo* efficacy of SP in the treatment of children between the ages of 6 and 59 months with uncomplicated malaria using WHO standard methodology [9]. Over the past 10 years, the recommended follow-up time in these studies has changed from 14 days to 42 days and in some cases to 63 days, to account for late treatment failures and to demonstrate the prophylactic effect of antimalarials. Since the WHO recommends treatment of uncomplicated malaria with an artimisinin-based combination therapy (ACT), it is no longer acceptable in most countries to carry out *in vivo* efficacy studies of SP used alone for treatment of uncomplicated malaria in children.

*In vitro* methods for measuring antimalarial drug resistance are being proposed to enable investigation of parasite resistance to the individual components of ACTs [10] and will be a useful adjunct to *in vivo* studies. However, these methods are only available in a few centres and no data are available from the sites of the SP-IPTi studies.

If *in vivo* studies cannot ethically be carried out and *in vitro* assays are unavailable then assessment of the level of resistance must rely on studies of the frequency of molecular markers of SP resistance. The mechanism of action of SP is well documented and point mutations at codons 16, 50, 51, 59, 108 and 164 in the *dhfr* gene [11], [12] confer resistance to pyrimethamine while mutations at codons 436, 437, 540, 581 and 613 of the *dhp*s gene [13], [14] confer resistance to sulfadoxine. There is a non-linear relationship between the number of mutations and resistance. However, the presence of three *dhfr* mutations (*dhfr* triple: N51I, C59R, S108N) and two *dhps* mutations (*dhps* double: A437G, K540E) in *Plasmodium falciparum* parasites studied prior to treatment is a significant predictor of SP treatment failure [15], [16], [17]. A recent meta analysis of SP *in vivo* studies and mutations showed a significant increase in the risk of therapeutic failure associated with the *dhfr* triple mutation (Day 28 OR 3.1 95% CI: 2.0–4.9) and with the *dhfr-dhps* quintuple mutation (Day 28 OR 5.2 95% CI 3.2–8.8) [18].

In this paper we characterise the relationship between protective efficacy of SP- IPTi, molecular markers of SP resistance and estimates of SP resistance measured by *in vivo* efficacy studies. By combining data from 7 randomised trials which evaluated the efficacy of IPTi against clinical malaria with contemporaneous data on resistance and the frequency of mutations in the *dhfr* and *dhps* genes in the study areas, we provide estimates of SP-IPTi protective efficacy at different levels of SP resistance with the aim of providing evidence towards defining the level of resistance at which SP-IPTi no longer provides a clinical protective effect.

## Methods

### a) Data Sources

Randomised placebo controlled (RCT) trials of IPTi were identified by literature search using the strategy shown in supplement file S1. Trials were only selected if they were RCT s, all participants were infants (children <1 year old) and SP treatment doses were given at the time of vaccination. Seven SP-IPTi trials were identified, all were undertaken in Sub-Saharan Africa between 1999 and 2008. The dates, locations and dosing strategies of these 7 studies are summarized in Table 1. There was substantial variability in the study design of the 7 trials, 3 out of 7 giving doses at 3, 9 and 15 months of age [2], [4], [7], 2 giving doses at 2,3 and 9 months of age [5], [6] and 2 giving doses at 3,4 and 9 months of age [1], [3] with one of these giving a further dose at 12 months [1]. The number of treatments with SP-IPTi given will affect protective efficacy up to 12 months of age. Thus, whilst these data are of clinical importance, comparisons of 12 month efficacy between trials should be viewed with caution. Although 12 month data are shown we attach more weight to the relationships between the protective efficacy in the 35 day post dose prophylactic period following administration of the 3 and 9 month doses of SP-IPTi which were given in all trials. Because only summary data were available, it was not possible to adjust our estimates to properly account for variations in the exact age at the time of administration of these doses or other factors such as ITN coverage between the studies. Summary data were supplied by the Statistical Working Group of the IPTi consortium (www.IPTi-malaria.org summarised in [19] ) and from a recently published IPTi study [6]. Unadjusted protective efficacies (PE) were estimated using the equation PE = 1−(incidence in the intervention group/incidence in the placebo group) and are shown in Table 2.

**Table 1:** IPTi study sites and corresponding studies selected for comparative studies of *in vivo* resistance and the frequency of *dhfr* and *dhps* mutations associated with resistance to SP.

IPTi Study			Efficacy paper reference used						Molecular data reference used				
Site	Study Period	Age at IPTi doses (months)	Reference	Site	Estimated Km from IPTi trial	Year	Design	Number of study participants	Reference	Site	Estimated Km from IPTi trial site	Year	Design
Ifakara, Tanzania [5]	1999–2000	2,3,9	[25]	Ifakara Town	0	1999	WHO 28 day pcr uncorrected	117	Malisa et al, submitted	Ulanga, Kilombero	13–35	2000	Community cross sectional
Manhica, Mozambique [3]	2002–2004	3,4,9	[34]	Manhica Town	0	2001	WHO 28 day pcr uncorrected	89	[35]	Manhica	0	2002	Malaria cases in IPTi study
Navrongo, Ghana [1]	2000–2002	3,4,9,12	[36]	Navrongo	0	2003	WHO 14 day pcr uncorrected	116	Chandramohan et al in prep	Navrongo	0	2003	WHO Efficacy study
Lambaréné, Gabon [6]	2004–2005	3,9,15	Mombo-Ngoma in prep	Lamberene	0	2007	WHO day 28 pcr corrected	26	Mombo-Ngoma in prep	Lamberene	0	2007	WHO Efficacy study
Kumasi, Ghana [2]	2003–2005	3,9,15							[37]	Bodomas, Ashanti	0	2003	Community cross sectional
Tamale, Ghana [4]	2003–2005	3,9,15	[38]	Bulpeila Health Centre	0	2002	WHO day 28 pcr corrected	126	[39]	Tamale	0	2002	WHO Efficacy study
Korogwe, Tanzania [6]	2004–2008	2,3,9	[26]	Hale Health Centre	27	2007	WHO day 28 pcr corrected	67	[26]	Hale	27	2007	WHO Efficacy study

**Table 2:** Protective efficacy of IPTi with SP, *in vivo* efficacy of SP in under 5 year olds and frequency of genetic markers of SP resistance at the seven study sites.

	IPTi efficacy (95% CI)		*In vivo efficacy*			Frequency of mutations		
Trial Site	35 day post dose prophylactic effect after 3 month dose, %	35 day post dose prophylactic effect after 9 month dose, %	Up to 12 months of age, %	Day 14 ACPR, %	Day 28 ACPR, %	DHFR triple, %	DHPS double, %	Quintuple, %
Ifakara, Tanzania [5]	77.8 (20, 100)*	91.1 (74, 100)	57.5 (43, 69)	68.9	59.8	24.4	8.2	3.6
Manhica, Mozambique [3]	57.5 (22, 80)	65.2 (39, 83)	15.4 (2, 27)	78.6	Not reported	81	56	52.9
Navrongo, Ghana [1]	75.8 (56, 89)	79.0 (70, 86)	30.0 (22, 37)	77.6	Not reported	44	1	0
Lambaréné, Gabon [7]	74.8 (−51, 100)**	71.5 (−25, 100)****	22.5 (−17, 50)	79	46	98	4	4
Kumasi, Ghana [2]	82.0 (66, 94)	47.6 (19, 68)	20.7 (8, 31)			61	1	1.3
Tamale, Ghana [4]	65.7 (19, 91)	83.4 (66, 95)	32.1 (20, 42)	86	72.2	47	1	0.8
Korogwe, Tanzania [6]	1*** (−397, 100)	69.6 (15, 95)	−10.2 (−52, 20)	38.8	17.7	96.4	90	89.2

*9 cases in placebo group,

**4 cases in placebo group,

***3 cases in placebo group,

****7 cases in placebo group.

Study sites varied in transmission intensity, the lowest being in Gabon [7] with an incidence of 0.22 cases of malaria per person year at risk (PYAR) and the highest being Kumasi in Ghana [2] with an incidence of 1.29 cases PYAR. Patterns of transmission also varied between sites with 2 sites supporting perennial transmission [2], [5], 4 sites perennial with seasonal peaks [3]–[7] and one site with highly seasonal transmission [1]. ITN coverage also varied between sites (5 studies <30% coverage [1]–[4], [7], and 2 sites >60% [5], [6]).

A literature review was undertaken to identify data for standard *in vivo* efficacy studies of SP in children under the age of 5 years and studies of mutations in *dhfr* and *dhps* genes conducted in locations near to the above trials in time and place. Search terms used in the review are shown in supplement file S1. Where no publications were found, researchers in areas where SP-IPTi had been conducted were contacted to acquire unpublished data. *In vivo* efficacy and mutation data were included if they were sampled within 2 years from the time of the SP-IPTi study and within a 50km radius. Variables extracted were study site, estimated distance from IPTi study site, year of study, study design, number of participants, day 14 and/or day 28 Adequate Clinical and Parasitological Response (ACPR) where available and frequency of mutations in *dhps* and *dhfr* genes (see tables 1 and 2).

### b) Statistical Methods

Univariate exploratory analyses of the relationship between the protective efficacy as measured in the trials and the measure of resistance (day 14 ACPR, frequency of triple *dhfr* mutation, frequency of *dhps* double mutation and their combination in a quintuple mutation) were first undertaken using non-parametric methods (Spearman's rank correlation) using Stata v10.1. However, these only assess the crude relationship between summary measures, and they will tend to under-estimate the strength of the association when the outcome measures have not been estimated precisely. In addition, they cannot easily be extended to predict protective efficacy at sites with different resistance profiles. To do this, we developed a simple non-linear probabilistic model of the relationship between mutation frequencies in the *dhfr* and *dhps* genes and the protective efficacy of SP-IPTi. The details of the development of the model are shown in supplement file S2. Briefly, the model takes into account the effect of each combination of mutations on PE, i.e. the effect of wild type parasites, the effect of the mutant haplotypes in the *dhfr* and *dhps* genes independently and when they are combined in the quintuple genotype.

To calculate the length of protection of SP-IPTi we used the two full data sets available (Navrongo, Ghana and Korogwe, Tanzania) [1], [6]. We examined the relationship between duration of protection after a dose of SP-IPTi and markers of SP resistance. The methodology used has been previously published [20], [21]. In short, person-time at risk after a particular dose of IPTi was divided into strata to allow calculation of protective efficacy in discrete time periods after IPTi. Random-effects Poisson regression was used to calculate protective efficacy in each time stratum. In this analysis we use a shorter time period (21 days *vs* 28 days) in order to demonstrate differences in the period of prophylaxis.

## Results

Six studies measuring *in vivo* efficacy of SP in children under the age of 5 years were found which met the inclusion criteria (Table 1). One site, Kumasi in Ghana, had no contemporaneous data on SP efficacy. ACPR was reported for the day 14 endpoint in all 6 studies and for day 28 in 4 of the 6 studies. Molecular PCR correction for re-infection and recrudescence was carried out in 3 of the 6 studies (Table 1).

Seven studies which met the inclusion criteria and tested for *dhfr* and *dhps* mutations were found (Table 1). All seven studies were undertaken within 50km of the IPTi studies and 5 of the 7 took place at the same site. Two were community cross sectional studies, 1 was from a study of clinical cases in an IPTi study and 4 were obtained at enrolment into standard *in vivo* efficacy studies. All studies reported results on the following codons; *dhfr* codons 51, 59 and 108 and for *dhps* codons 436, 437 and 540, and these were therefore used for further analyses. Six studies reported on mutations at codon 164 on the *dhfr* gene (none found mutations at this codon) and 4 studies reported on mutations at codons 581 and 613 on the *dhps* gene.

Table 2 summarises the three measures of protective efficacy of IPTi (namely the 35 day post dose prophylaxis effect of the 3 and 9 month doses of SP and the protective efficacy of SP IPTi to 12 months of age) for the seven trials, and the 14 and 28 day ACPR and frequency of *dhfr* triple and *dhps* double mutations found in studies conducted nearby in time and location.

### Univariate associations between markers of resistance and protective efficacy of SP-IPTi

**Figure 1:**
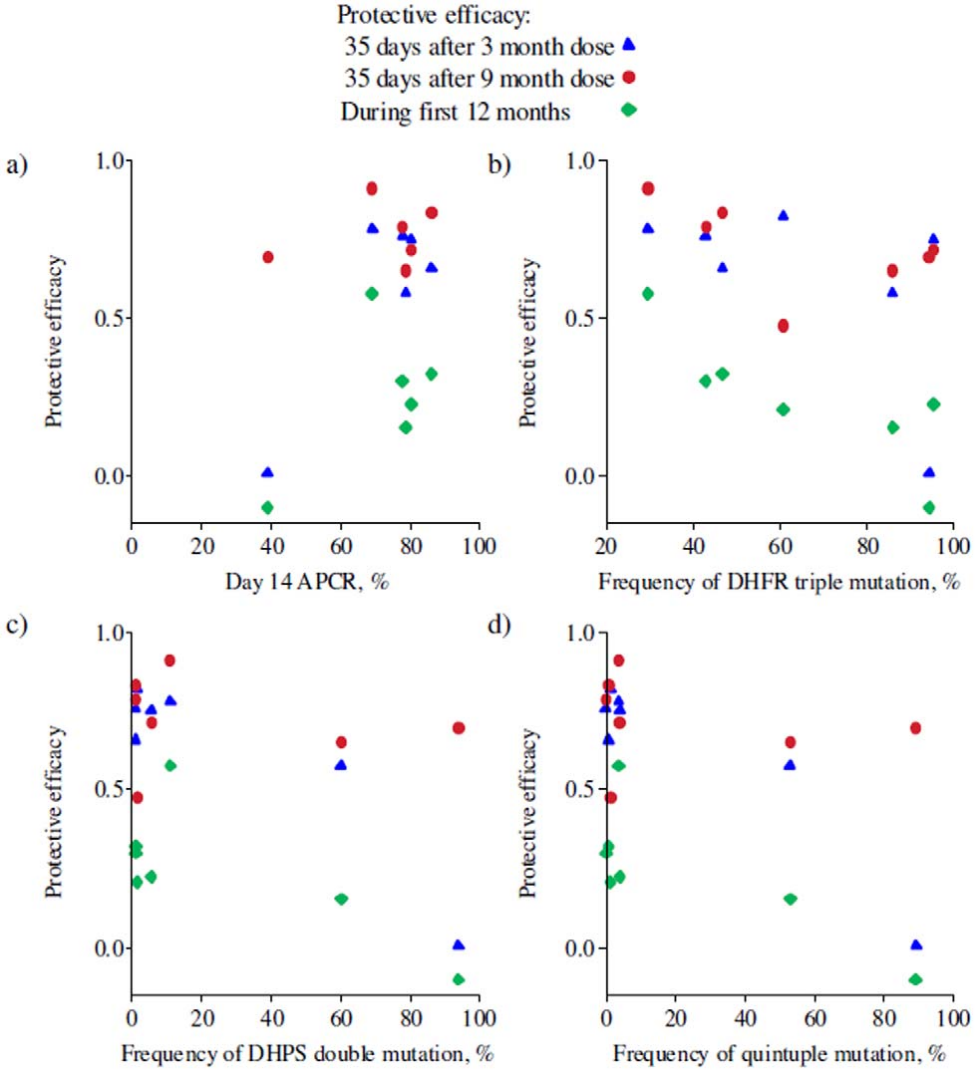
Relationship between protective efficacies of IPTi for the seven trial sites plotted against markers of resistance. (a) the 14 day ACPR (rank correlation = 0.03 (p = 0.96) for the 3 month dose, 0.09 (p = 0.87) for the 9 month dose and 0.26 (p = 0.62) over 12 months); (b) the frequency of the *dhfr* triple mutation (rank correlation = −0.54 (p = 0.22) for the 3 month dose, −0.61 (p = 0.15) for the 9 month dose and −0.75 (p = 0.05) over 12 months); (c) the frequency of *dhps* double mutation (rank correlation = −0.46 (p = 0.29) for the 3 month dose, −0.32 (p = 0.48) for the 9 month dose and −0.61 (p = 0.15) over 12 months); (d) the frequency of the *dhfr/dhps* quintuple (rank correlation = −0.66 (p = 0.16) for the 3 month dose, −0.26 (p = 0.62) for the 9 month dose and −0.71 (p = 0.11) over 12 months).

Figure 1 shows the relationship between four measures of SP resistance and the protective efficacy of SP-IPTi against incidence of malaria during the 35 days after administration of the 3 and 9 months doses of SP-IPTi and up to 12 months of age. The measures of SP resistance are: a) the day 14 failure rate from *in vivo* efficacy studies; b) the frequency of the *dhfr* triple mutation; c) the frequency of the *dhps* double mutation; and d) the frequency of the quintuple *dhfr/dhps* mutation. There is some indication that as resistance increases protective efficacy declines, as indicated by the negative rank correlations in all comparisons and there is a significant negative rank correlation between frequency of the triple *dhfr* genotype and 12 month protective efficacy −0.75 (p = 0.05). It should be noted that the 12 month protective efficacies are not directly comparable between trials due to differences in both the timing and number of doses of IPTi delivered as well as the length of follow-up.

### Estimates of the relationship between mutation frequency and protective efficacy

**Figure 2:**
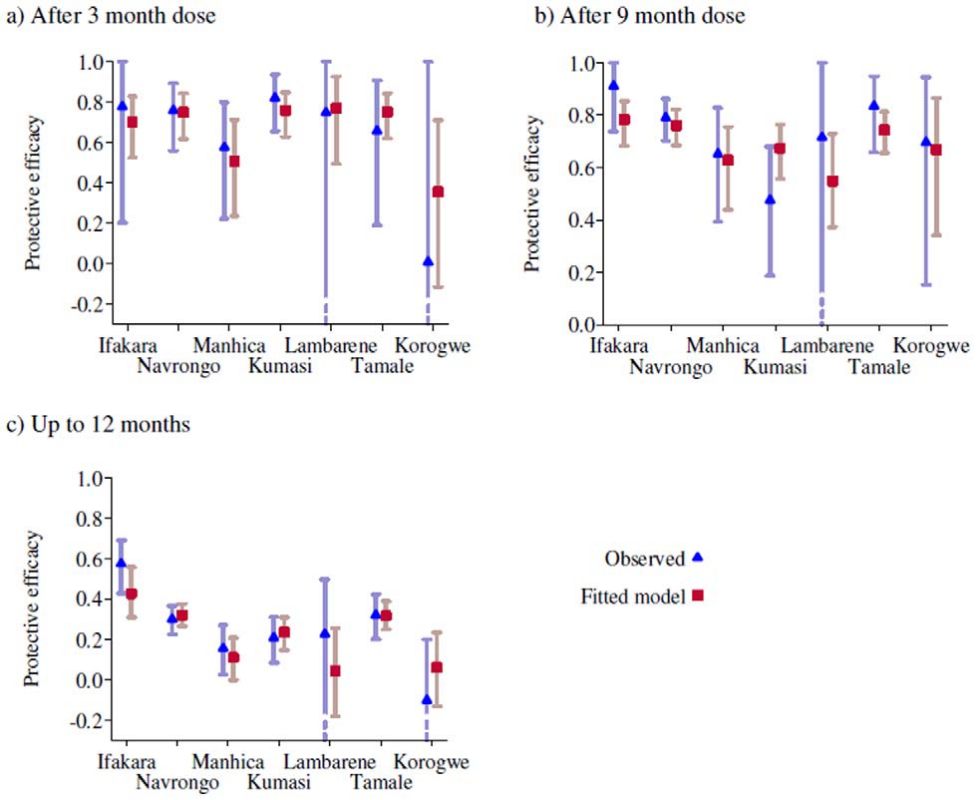
Observed and fitted protective efficacies of IPTi for the seven trial sites. a) during 35 days after a dose at 3 months; b) during 35 days aftera dose at 9 months; and c) up to 12 months of age. Model predictions are based only on the frequency of the *dhfr* triple mutation, *dhps* double mutation and combined *dhfr* and *dhps* quintuple mutation and do not adjust for any other differences between the trials or the trial sites.

Figure 2 shows the fit of the model expressing the relationship between mutation frequency and protective efficacy in each of the 7 sites for the 35 days after the 3- and 9-month doses and up to 12 months of age. For all outcomes there is close agreement between the model and the data to which it is fitted, demonstrating that there is a strong relationship between protective efficacy and the frequency of resistance mutations in the population. For the 3 month dose, the model-predicted efficacy is somewhat higher than that observed in Korgowe, although the confidence interval for the data estimate is wide and the observed protective efficacy is within the 95% credible interval predicted by the model. There is greater discrepancy for the 9 month estimates with higher observed protective efficacies in Ifakara , Lambaréné and Tamale compared to those predicted by the model and lower observed PE in Kumasi than predicted by the model. For the 12 month outcome the model predictions for the Ifakara and Lambaréné datasets are substantially lower than that observed whereas the model predicted a higher efficacy for the Korogwe site. Thus, for the 9 month dose and up to 12 months of age data it appears that factors other than the mutations investigated were influencing protective efficacy. This has been explored in two modelling exercises [22], [23].

Table 3 shows the estimated protective efficacy for 35 days following the 3 and 9 month doses and up to 12 months of age for wild type (no mutations) and each of the three mutation combinations. The estimates for the *dhps* double mutation alone are uncertain (as demonstrated by the wide credible intervals) since almost all samples which contained the double mutation also contained the triple *dhfr* mutation. However, because the frequency of the double *dhps* mutation alone is low, this uncertainty has little impact on the predicted efficacies for each site. Our parameter estimates suggest that IPTi will be significantly less efficacious in a population with 100% prevalence of the triple *dhfr* mutation than in one where there are no mutations, with the difference between the protective efficacy in the latter and former cases estimated as −0.08 (95% credible interval (CrI): −0.53, 0.45) after the 3 month dose, 0.42 (95% CrI: 0.03, 0.65) following the dose at 9 months and 0.60 (95% CrI 0.11, 1.12) up to 12 months of age. It was not possible from these data to provide sensible estimates of the relative efficacy with the double mutation compared to either no mutations, or to the triple mutation, or with the quintuple mutation compared to the triple alone, because of a lack of power and hence precision.

**Table 3:** Estimated protective efficacy against malaria for 35 days post 3 and 9 month dose (posterior median and 95% credible interval) and up to 12 months of age of SP IPTi when the parasite population has no resistance mutations, all carry the *dhfr* triple mutation, all carry the *dhps* double mutation or all carry the quintuple mutation (both the *dhfr* triple and *dhps* double mutations).

	Mutations present	Estimated PE (%)	95% CrI	
After dose at 3 months	None	73.0	41.8	97.4
	Just *dhfr* triple	80.5	48.2	98.6
	Just *dhps* double	38.0	−41.0	97.1
	Both	31.8	−22.5	73.5
After dose at 9 months	None	94.2	74.8	99.8
	Just *dhfr* triple	52.6	32.5	74.0
	Just *dhps* double	50.1	−34.4	97.9
	Both	67.8	30.1	91.5
Up to 12 months of age	None	60.0	34.3	87.8
	Just *dhfr* triple	0.1	−26.3	25.5
	Just *dhps* double	67.5	−19.4	98.7
	Both	1.8	−20.6	23.4

**Figure 3:**
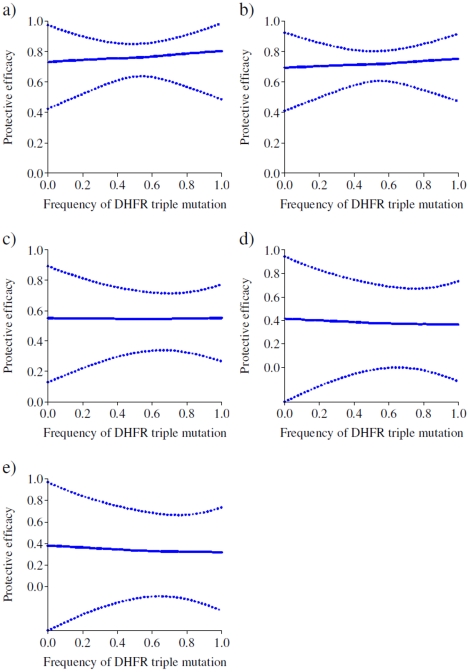
Expected protective efficacy of SP- IPTi of the 3 month dose at different frequencies of the *dhfr* triple mutation in settings of different frequencies of the *dhps* double mutation. (a) 0%, (b) 10%, (c) 50%, (d) 90% and (e) 100% frequency of the *dhps* double mutation.

**Figure 4:**
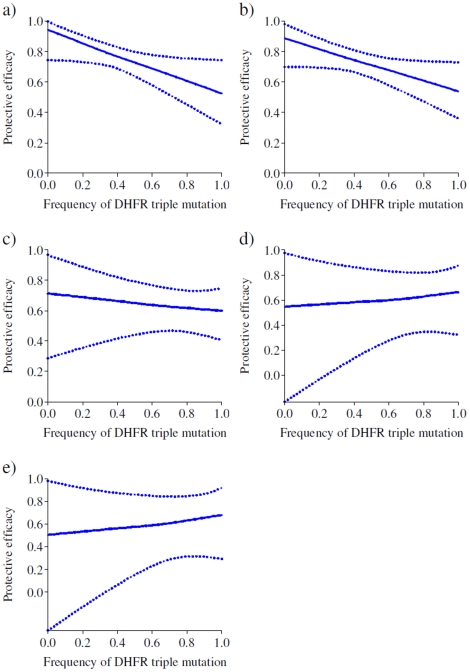
Expected protective efficacy of SP- IPTi after the 9 month dose at different frequencies of the *dhfr* triple mutation in settings of different frequencies of the *dhps* double mutation. (a) 0%, (b) 10%, (c) 50%, (d) 90% and (e) 100% frequency of the *dhps* double mutation.

**Figure 5:**
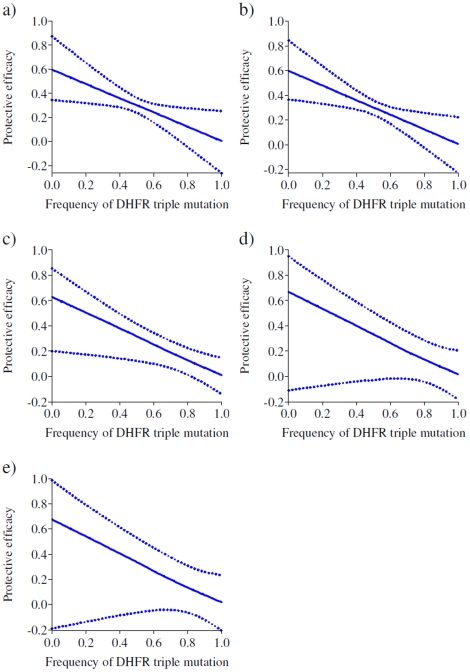
Expected protective efficacy of SP- IPTi up to 12 months of age at different frequencies of the *dhfr* triple mutation in settings of different frequencies of the *dhps* double mutation. (a) 0%, (b) 10%, (c) 50%, (d) 90% and (e) 100% frequency of the *dhps* double mutation.

Figures 3, 4 and 5 show the expected 35 day protective efficacy of doses of SP-IPTi given at 3 and 9 months of age and up to 12 months of age respectively for different levels of frequency of both *dhfr* triple and *dhps* double mutations. There is little observable relationship between protective efficacy and the frequency of the *dhfr* triple mutation following the 3 month dose regardless of the frequency of the *dhps* double mutations (Figure 3), most likely due to a lack of power given the small number of events in this period. However, there is a significant decrease in protective efficacy after the 9 month dose with increasing frequency of the *dhfr* triple mutation at low frequencies of the *dhps* double mutation (Figure 4 a and b, 0% and 10% frequency respectively). For higher frequencies in the double *dhps* mutation, the relationship between the *dhfr* triple frequency and protective efficacy is less apparent (Figure 4 c–e). However, this is most likely due to the imprecision of these estimates (as shown by the wider credible intervals) as in practice the *dhps* double mutation is almost always accompanied by a high frequency in the *dhfr* triple mutation. The predicted protective efficacy up to 12 months shows a decrease for all frequencies of the *dhps* double mutation (Figure 5), although again the relationship is uncertain for higher *dhps* double frequencies, and as noted the relationship for the 12 month outcome may be biased due to variations in study design.

### Results of length of protection analysis

**Figure 6:**
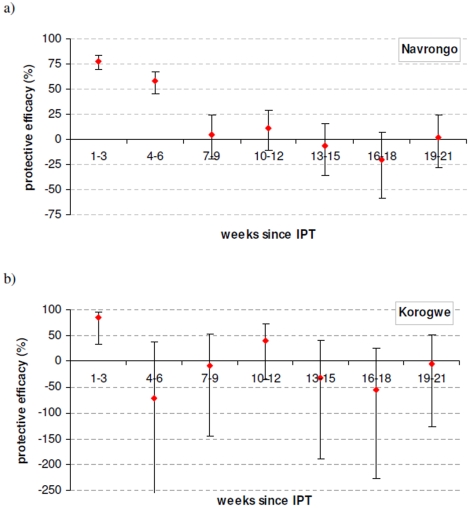
Length of protection of SP- IPTi in 2 settings. a) low resistance to SP and b) high resistance.

In Navrongo Ghana, where *P. falciparum* resistance to SP is low, the period of protection post IPTi dose extended to 42 days. In contrast, in the high resistance setting of Korogwe, Tanzania, the period of protection was reduced to 21 days with an increased risk of malaria, shown by a non-significant negative protective efficacy during the second 21 day period (Figure 6 a and b).

## Discussion

Our results suggest that there is a reduction in the protective efficacy of SP-IPTi with increasing molecular markers of SP resistance in contrast to our previous analysis with fewer studies that demonstrated no association between day 14 ACPR and resistance [24]. We previously stated that the site with the highest protective efficacy for SP-IPTi, Ifakara, also had the highest resistance using ACPR as a measure of resistance [25]. This *in vivo* study was carried out immediately prior to the IPTi study in the same study site but did not collect molecular data. In our search for data on molecular markers of resistance we found a study that took place around the same time and was within 20km of the IPTi study site. The results from this study show that the Ifakara site had the lowest frequency of *dhfr* and *dhps* mutations. Explaining these contrasting observations is difficult. One possibility is that the IPTi and *in vivo* study were located in a semi-urban site which had a higher density of drug shops than in the rural villages where the molecular studies were done and easier access to the district hospital thus resulting in greater drug pressure and higher resistance levels as reflected by the high ACPR. Another possibility is that the high failure rate seen in the *in vivo* study [25] was due to re-infections. However, this is unlikely because the incidence rate reported in the IPTi study was low-moderate (0.36 episodes PYAR) [5] and, if the parasites were very sensitive as found with the molecular data, then we would expect SP to have offered prophylaxis beyond 28 days. Hence, it is unclear which data best represent resistance levels for the Ifakara site.

We were able to only show a single, borderline statistically significant association between the measures of resistance to SP that we examined and protective efficacy of SP-IPTi using simple exploratory analyses with the few data points available. However, by fitting a simple probabilistic model of both *dhfr* triple and *dhps* double mutations, which better represents the underlying relationship between the mutations and protective efficacy, we were able to obtain a reasonable fit to the data and thus demonstrate a strong relationship between the level of resistance mutations (notably *dhfr* triple mutation) and protective efficacy. For low levels of the *dhps* double mutation, there was a significant decrease in the efficacy of IPTi 35 days post the 9 month dose with increasing frequency of the *dhfr* triple mutation. However, as the frequency of the *dhps* double mutants increased the estimates of the relationship between *dhfr* triple and protective efficacy became less precise. This supports the biological plausibility of the model as *dhfr* mutations first appear followed by *dhps* mutations. Once the frequency of the *dhfr/dhps* quintuple mutation rises, selection for more resistant haplotypes takes place, as in the case of Korogwe, where the quintuple mutation reached saturation and there was a high frequency of a sixth mutation, the *dhps* 581G mutation [26].

The analysis was limited by the scarcity of data points. In 3 of the 7 studies there were fewer than 10 cases of malaria in the placebo group during the 35-day period following the 3-month dose and 1 of the 7 studies had simiarly few cases after the 9 month dose. There was also a lack of variation in the frequency of molecular mutations with only 2 sites having frequencies of the *dhfr/dhps* quintuple mutation above 4%, and all sites with a high frequency of the *dhps* double mutation also had a high frequency of the *dhfr* triple mutation. Because of this, the model is unable to provide sensible predictions of the protective efficacy for the quintuple mutation, with the central estimate obtained being higher than that for the *dhfr* triple and *dhps* double individually at the 9 month dose. This is not consistent with our biological understanding of the mechanism of resistance to SP or evidence from *in vivo* studies [18] but simply reflects a lack of data rather than an underlying problem with the model. This lack of data meant that we were unable to undertake analyses of individual mutations including analysis of additional mutations to the quintuple, for example the *dhps* 581 G mutation found in more than 50% of samples at the Korogwe site [26] that might make parasites even more resistant to SP than those carrying the quintuple mutation alone.

The use of the 12 month efficacy results for comparing sites should be interpreted with caution as 3 out of the 7 studies gave two treatments with SP during the first year of life whereas the remaining 4 gave three doses. In addition, 4 of the 7 studies provided 10 months of observation in the first year and 3 had only 9 months. Both these factors would have affected the protective efficacy of SP-IPTi resulting in biased estimates of the association between protective efficacy and resistance-conferring mutations. The results using the 12 month endpoint show an apparent association between protective efficacy and both the *dhfr* triple and the *dhfr/dhps* quintuple mutations, and although this is biologically plausible, this association suffers from bias. Thus, in an attempt to compare like with like we chose to examine the post treatment prophylactic periods. Whilst the 35-day post-dose protective efficacy is meaningful in terms of comparing SP efficacy between settings with different levels of resistance, for clinical practice a longer period is more relevant. Unfortunately it is not straightforward to translate an expected protective efficacy at day 35 post 3 and 9 month dose predicted by the model at different frequencies of mutations to an equivalent 12 month protective efficacy. For example, in Korogwe, Tanzania the 35 day protective efficacy after the 9 month dose was 70% but protective efficacy up to 12 months of age was −6% [6]. Thus further studies or modelling are required to assess the relationship between the *dhfr* mutation frequencies and protective efficacy over this longer period.

Our analysis of the length of the period of prophylaxis in the low and high resistance settings of Navrongo, Ghana and Korogwe, Tanzania respectively provide further insight into the mechanism underlying the relationship between mutation frequency and protective efficacy. In this analysis, we have shown that increasing SP resistance shortens the period of prophylaxis. This is explained biologically by parasites with mutations requiring greater minimum inhibitory concentrations of SP to kill or suppress the parasites [27]. Thus, SP can be efficacious while levels of SP remain high in blood, evident from the high protective efficacy (>55%) during 35 days after the 3 and 9 month doses in 6 out of the 7 trial settings including those with high resistance to SP [3], [6]. Observation of the length of prophylaxis in 4 of the 7 studies shows there is a high level of protection in the 2–6 weeks after a dose of SP-IPTi with protection declining as drug levels decrease in settings of low resistance [20], [28] and to a negative protective efficacy in the very high resistance setting [21].

Is there a measure of SP resistance that we can use to determine SP efficacy for IPTi? Our study suggests that an *in vivo* study in asymptomatic infants specifically designed to look at the prophylactic effect of antimalarials might be more informative if it looked at the duration of the post dose prophylactic effect rather than at the protective efficacy to a certain time point, such as 35 days post dose used in this analysis. If *in vivo* studies cannot be done then an assessment of molecular markers should be made. Maps of the occurrence of the key *dhfr* and *dhps* mutations across Africa have recently been published [26], [29], [30] (also see www.drugresistancemaps.org). These show that the highest rates of mutations of *dhfr* and *dhps* genes are in East and Southern Africa where SP use has been the highest. Thus, on the basis of our model we would predict that that IPTi with SP would currently be more effective in West and Central Africa than in East and Southern Africa where both *dhfr* and *dhps* mutations are more frequent. However, future expansion or contraction of SP resistance may alter this situation and thus continued monitoring of drug resistance in malaria endemic countries is essential. Where the frequency of the quintuple mutation rises above 60% further resistant haplotypes such as the *dhfr* 164 L in Uganda and Rwanda [30], [31] and the *dhps* 581 G in Tanzania, Uganda and Rwanda [26], [30], [31], [32] are selected. It is highly unlikely that SP- IPTi will have a significant protective effect in areas in which these mutations are common, as demonstrated in northern Tanzania [6]. Thus, additional measurement of the frequency or prevalence of the quintuple mutation alongside measurements of both the *dhfr* and *dhps* mutations may guide policy makers in deciding where to implement SP-IPTi. More recently, an additional Technical Expert Group was convened by the WHO that reviewed data on SP-IPTi protective efficacy and SP resistance that included data from this paper. At this meeting a consensus was reached that the cut-off for implementation for SP-IPTi should be a prevalence of the quintuple *dhfr/dhps* mutation of 50%. As it is not always possible to measure all mutations the recommendation was to use the *P falciparum dhps* 540 mutation as a marker for the quintuple mutation and where this was greater than 50% SP-IPTi should not be implemented. In addition, the final WHO policy document states that in situations where a National-scale implementation may not be feasible due to varying levels of the *dhps* 540 mutation, IPTi may be implemented at a Provincial or District scale, targeting areas with *dhps* 540 mutation prevalence ≤50% [33].

## Supporting Information

File S1 Literature search strategy
(0.03 MB DOC)

File S2 Development of the model. Development of a simple probabilistic model to examine the relationship between mutations in the dhfr and dhps genes and protective efficacy of SP-IPTi.
(0.07 MB DOC)
