# Supplementary material for: Protective Efficacy of Intermittent Preventive Treatment of Malaria in Infants (IPTi) Using Sulfadoxine-Pyrimethamine and Parasite Resistance
Source: PLoS One. 2010 Sep 7;5(9):e12618. doi: 10.1371/journal.pone.0012618 (PMC2935388; doi:10.1371/journal.pone.0012618)
Supplement: File S1 — Literature search strategy (0.03 MB DOC) [file pone.0012618.s001.doc]

**Supplement S1 Literature search strategy for “Protective efficacy of Intermittent Preventive Treatment of malaria in infants (IPTi) using sulfadoxine- pyrimethamine and parasite resistance”:**

To identify all randomized placebo control trials of IPTi using sulfadoxine- pyrimethamine (SP) we used the following search strategy in the National Library of Medicine search engines, Pubmed and Medline. We used the following terms as free text; intermittent, malaria, treatment, sulfadoxine, sulphadoxine, and pyrimethamine, Fansidar and infants.

A further literature search was done using the National Library of Medicine search engines, Pubmed and Medline. For the search for *in vivo* studies we selected articles published in English and we used search terms of the country and region in which the IPTi studies were carried out plus sulphadoxine, sulfadoxine, pyrimethamine, Fansidar, *in vivo* and malaria. For the molecular studies we selected articles published in English and search terms of the country and region in which the IPTi studies were carried out plus *dhfr* , *dhps* sulphadoxine, sulfadoxine, pyrimethamine, Fansidar, prevalence, malaria and resistance as free text. Studies were not restricted to randomized controlled trials for the molecular studies.

We read each abstract and assessed whether or not the article was suitable. Hardcopies of papers matching the criteria were obtained and read fully. Articles were only accepted as IPTi efficacy studies if they reported a study that was a randomized placebo controlled trial of infants and doses of IPTi were given with EPI vaccinations. For the *in vivo* and molecular studies were included if they reported data that was collected within 2 years of the data collection of the corresponding IPTi study and within 100km of the IPTi study.
