# Supplementary material for: Protective Efficacy of Intermittent Preventive Treatment of Malaria in Infants (IPTi) Using Sulfadoxine-Pyrimethamine and Parasite Resistance
Source: PLoS One. 2010 Sep 7;5(9):e12618. doi: 10.1371/journal.pone.0012618 (PMC2935388; doi:10.1371/journal.pone.0012618)
Supplement: File S2 — Development of the model. Development of a simple probabilistic model to examine the relationship between mutations in the dhfr and dhps genes and protective efficacy of SP-IPTi. (0.07 MB DOC) [file pone.0012618.s002.doc]

**Supplement S2 Development of a simple probabilistic model to examine the relationship between mutations in the *dhfr* and *dhps*  genes and protective efficacy of SP-IPTi.**

Suppose that the incidence of an outcome (e.g. clinical episode of malaria) is $\lambda_{P}$ in the placebo group and $\lambda_{T}$ in the intervention group. Then the protective efficacy (PE) of the intervention is defined as:

$$PE=1-\frac{\lambda_{T}}{\lambda_{P}}$$

Let the hypothetical incidence in the intervention arm be$\beta_{i}\lambda_{P}$, where *i* = 0 if all parasites have neither mutation, *i* = 1 if all parasites carry just the triple *dhfr* mutation, *i* = 2 if all parasites carry just the double *dhps* mutation, and *i* = 3 if all parasites carry both, i.e. the *dhfr-dhps*  quintuple mutation. The protective efficacy is$PE_{i}=1-\beta_{i}$. We would expect$0\leq PE_{i}\leq1$, with $PE_{0}$ the largest and possibly $PE_{3}$ the smallest.

Let the proportions of parasites circulating in a population which have neither, just the triple *dhfr*, just the double *dhps,* or *dhfr-dhps* quintuple mutation be $p_{0}, p_{1}, p_{2}\text{ and }p_{3}$ respectively. Ignoring any dependence between the outcomes of successive infectious challenges, the actual incidence in the intervention group should be

$$\lambda_{T}=\lambda_{P}\sum_{i=0}^{3} \beta_{i}p_{i}$$

and hence the PE with this parasite background should be

$$PE=\sum_{i=0}^{3} PE_{i}p_{i}$$

We model the number of events in each group as a Poisson distribution; to allow for unobserved factors which raise or lower the overall incidence in each study, we estimate a separate $\lambda_{P}$ for each site.

We denote the total proportion of parasites in the data with the triple *dhfr* or double *dhps* mutations by $q_{1} \text{and} q_{2}$ respectively, and let be the proportion out of those with the double that also have the triple (i.e. the quintuple mutation). Then -

$$\begin{matrix} p_{3}=q_{2}q_{32} \\ p_{1}=q_{1}-p_{3} \\ p_{2}=q_{2}-p_{3} \\ p_{0}=1-(p_{1}+p_{2}+p_{3}) \end{matrix}$$

Parameterising the model this way allows us to use all the available information even though not all samples were tested at both genes. As we are attempting to estimate the frequency of each haplotype in the parasite population we only considered samples which were unmixed or had a majority signal for a particular haplotype. In infections of more than one haplotype it is often not possible to determine haplotype due to the presence of both wild type and mutants being detected at the loci of interest. We estimated the parameters of the IPTi efficacy model jointly with the mutation frequencies so that the uncertainty in the latter is accounted for.

Our analysis was based on the summary data from 7 trials thus only 7 data points of which the commonest mutations were the triple *dhfr* and double *dhps* mutations. This meant that there was insufficient power to explore other combinations of mutations. We therefore chose the three combinations of mutations (triple *dhfr*, double *dhps* and both, the quintuple mutation) which we considered *a priori* on the basis of past studies to be the best candidate predictors.

Our parameter estimates are the medians of the posterior distributions, with 95% credible intervals (CrI). The posterior distribution was estimated using Markov Chain Monte Carlo (MCMC), implemented in C++ (Gilks, Richardson, & Spiegelhalter, 1996). MCMC chains consisted of a burn-in of 10,000 iterations followed by 1 million iterations, thinned by retaining every 20th iteration, on which inferences were based. For each outcome considered, five MCMC chains were run using a different random choice of initial values for all parameters. Convergence of the chains was assessed both by plotting the parameter values against iteration number for single chains, and by comparing the posterior median and credible intervals between chains. The latter quantities varied by 1% or less between chains.

We gave each $PE_{i}$ the following prior distribution with *a* = 0.75, meaning a prior probability of 0.75 that$0\leq PE\leq1$:

Each $\lambda_{P}$ was assigned an uninformative Gamma prior distribution with a mean of 1 and a shape parameter of 0.1. We took uniform prior distributions for $q_{1}$, $q_{2}$and , with the constraint that all the derived $p_{0}, p_{1}, p_{2}\text{ and }p_{3}$ must be between 0 and 1.
